# Evaluation of *Callistemon citrinus* Compounds to Reduce Brain Oxidative Stress in Rats Fed High-Fat-Sucrose Diet

**DOI:** 10.3390/metabo16010024

**Published:** 2025-12-25

**Authors:** Aram Josué García-Calderón, Oliver Rafid Magaña-Rodríguez, Luis Alberto Ayala-Ruiz, José Armando Hernández-Soto, Jonathan Saúl Piñón-Simental, Luis Gerardo Ortega-Pérez, Asdrubal Aguilera-Méndez, Patricia Ríos-Chávez

**Affiliations:** 1 Facultad de Biología de la, Universidad Michoacana de San Nicolás de Hidalgo, Morelia 58000, Mexico; 1208935X@umich.mx (A.J.G.-C.); oliver.rodriguez@umich.mx (O.R.M.-R.); 1232816G@umich.mx (L.A.A.-R.); 1719780C@umich.mx (J.A.H.-S.); jonathan.saul.simental@umich.mx (J.S.P.-S.); gerardo.ortega@umich.mx (L.G.O.-P.); 2 Instituto de Investigaciones Químico Biológicas de la, Universidad Michoacana de San Nicolás de Hidalgo, Morelia 58000, Mexico; amendez@umich.mx

**Keywords:** *Callistemon citrinus*, antioxidant system, limonene, phenolic acids, oxidative stress, inflammation

## Abstract

**Background**: The association between oxidative stress and inflammation in obesity motivates investigation of the effects of d-limonene, gallic acid, ellagic acid, p-coumaric acid, and their mixture, which are major compounds of *Callistemon citrinus*, on oxidative stress and inflammation in the brains of rats fed a high-fat-sucrose diet. This study aimed to identify the specific bioactive compounds in *C. citrinus* leaf extract responsible for its neuroprotective effects against diet-induced oxidative stress and neuroinflammation. **Methods:** Forty-eight male Wistar rats were randomly divided into eight groups (n = 6). Group 1 (control) received a standard diet, while group 2 received a high-fat, high-sucrose diet (HFSD). Groups 3, 4, 5, 6, 7, and 8 were also fed HFSD supplemented with *C. citrinus* extract, its main compounds, and a mixture of these compounds administered once daily via oral cannula for 23 weeks. The antioxidant and pro-inflammatory enzymes, along with oxidative biomarkers, were evaluated in the brains of the rats. **Results:** *C. citrinus* leaf extract and its four main components, both separately and together, modulated the activities of catalase, superoxide dismutase, glutathione peroxidase, and paraoxonase-1. They also affected levels of reduced glutathione while decreasing the amounts of advanced oxidative protein products, malondialdehyde, and 4-hydroxynonenal. Additionally, they decreased the activities of cyclooxygenase (COX-1 and COX-2), 5-lipoxygenase, xanthine oxidase, and myeloperoxidase in the brains of rats, despite a high-fat-sucrose diet. **Conclusions:** These results show that the main compounds in *C. citrinus* leaf extract are essential for its antioxidant and anti-inflammatory effects, which help protect against oxidative stress in the brains of rats on a high-calorie diet.

## 1. Introduction

Although dietary nutrients are essential substrates for cellular energy production, excessive consumption can lead to metabolic dysregulation. A diet high in saturated fatty acids can lead to obesity, which is linked to persistent low-grade inflammation. Due to the accumulation of visceral fat, there is increased expression of pro-inflammatory cytokines and enzymes involved in this process. One tissue affected by obesity is the brain, which is associated with neuroinflammation [1].

The state of low-grade inflammation in obesity also increases ROS production via mitochondrial dysfunction, increased NADPH oxidase activity, and lipid peroxidation [2]. At optimal levels, ROS plays a crucial role in various cellular processes, including defense against infectious agents, communication, and the maturation of cellular structures, among others. However, at high concentrations, they can be harmful because they react with various biomolecules, including lipids, proteins, carbohydrates, and nucleic acids (DNA and RNA), altering the structure and function of these macromolecules and causing damage at the cellular and tissue levels [3].

In pathological conditions such as overweight and obesity, an imbalance between energy intake and expenditure leads to abnormal or excessive fat accumulation, which can harm health [4]. Under these conditions, elevated blood glucose and lipid levels can increase ROS production. On the other hand, Jacques et al. [5] demonstrated how high sugar intake, besides contributing to obesity, can cause damage to the brain. Recently, there has been growing interest in identifying natural antioxidants as alternatives for preventing and treating diseases associated with oxidative stress. In this context, secondary metabolites in plants have attracted significant attention due to their safety and potential nutritional and therapeutic benefits for human health [6].

*Callistemon citrinus* is a plant native to Australia and belongs to the family Myrtaceae. It is considered an ornamental tree in Mexico; however, this species is used in traditional medicine in other countries [7]. Petronilho et al. [8] demonstrated the terpene profile of *C. citrinus* leaves and flowers. A previous study showed that the ethanolic extract from *C. citrinus* leaves exhibits significant antioxidant capacity against oxidative stress in obese animal models [9]. Similarly, d-limonene, 1,8-cineole, and α-terpineol, major terpenes found in *C. citrinus*, have shown anti-obesity, antioxidant, and anti-inflammatory effects in Wistar rats fed a high-fat-sucrose diet (HFSD), highlighting their beneficial effects against oxidative stress [10]. Ortega-Pérez et al. reported the presence of phenolic and flavonoid compounds in *C. citrinus* extract and in phytosomes loaded with *C. citrinus* [11].

Recently, Magaña-Rodríguez et al. [12] reported the beneficial effect of *C. citrinus* extract and phytosomes loaded with *C. citrinus* on oxidative stress in the brains of rats fed a high-fat-fructose diet for 16 weeks. Despite the numerous active compounds in *C. citrinus* extract, the specific ones responsible for its antioxidant and anti-inflammatory effects remain unclear. In this context, the current study aims to investigate the antioxidant and anti-inflammatory properties of the primary compounds (d-limonene, gallic, ellagic, p-coumaric acids, and their mixture) found in the *C. citrinus* extract on oxidative stress and inflammation in the brains of rats fed a high-fat-sucrose diet.

## 2. Materials and Methods

### 2.1. Equipment and Chemical Compounds

Spectrophotometer Thermo Spectronic model Genesys 10 Uv, brand Spectronic Unicam (Rochester, NY, USA), Büchi ^®^ Rotavapor ^®^ R-210 (Büci Labortechnik AG, in Flawil., Switzerland). R-(+)-limonene 97% (Cat No. 183164), gallic acid (Cat No G7384), ellagic acid hydrate (Cat No 372749), p-coumaric acid 98% purity HPLC (Cat No C9008), hydrogen peroxide, blue nitrotetrazolium chloride, riboflavin, methionine, NADPH, reduced glutathione, glutathione reductase, cumene, 4-nitrophenyl acetate, 5,5′-dithiobis (2-nitrobenzoic acid), 1-methyl-2-phenylindole, methanesulfonic acid, potassium iodide, hematin, N,N,N′,N′-tetramethyl-p-phenylenediamine dihydrochloride, arachidonic acid, orange xylenol, ferrous sulfate, butylhydroxytoluene, xanthine and tetramethylbenzidine and all other chemicals and reagents were of analytical grade of Sigma-Aldrich (St. Louis, MO, USA).

### 2.2. Plant Collection and Preparation of Ethanolic Extract

*C. citrinus* leaves were collected in Morelia, Mich., Mexico, in August 2023. The fresh leaves were macerated in 99.45% ethanol Sigma-Aldrich (Cat. No. E7023) at a 1:10 (*w*/*v*) ratio. The extract was left to settle at 20 ± 1 °C in the dark for five days. Subsequently, it was dried under vacuum using a rotary evaporator (Buchi R-210, Flawil, Switzerland) at 45 ± 1 °C and stored at 4 ± 2.8 °C until further use.

### 2.3. Experimental Design and High Fat-Sucrose Diet

Wistar male rats weighing 200–250 g were obtained from the animal laboratory at Universidad Michoacana de San Nicolás de Hidalgo. The animals were housed in cages at the vivarium of the Chemical-Biological Research Institute of the University of Michoacana of San Nicolás de Hidalgo. They were raised under standard conditions, with a 12-h light-dark cycle, an average temperature of 20 °C, a relative humidity of 60–70%, and free access to water and a balanced diet. The manipulation and experimentation of the animals were conducted in accordance with the Federal Regulation for the Care and Use of Laboratory Animals, issued by the Official Mexican Standard (NOM-062-ZOO-1999) of the Ministry of Agriculture of Mexico. The Animal Bioethical Committee of UMSNH approved the study on 12 January 2024, with approval code ID IIQB-CIBE-06-2024. Every effort was made to minimize animal suffering and decrease the number of animals used in the study. The choice of Wistar rats over other strains is based on their ease of handling, genetic uniformity, and ability to produce consistent results. The duration of the experiment was based on a previous study that involved 16 weeks of feeding a high-fat, high-fructose diet, which altered the rat brain’s antioxidant systems [12]. Additionally, Eroglu et al. [13] reported that after 26 weeks, an inflammatory process was observed in all brain regions, which was not fully present during a 13-week period.

**Figure 1:**
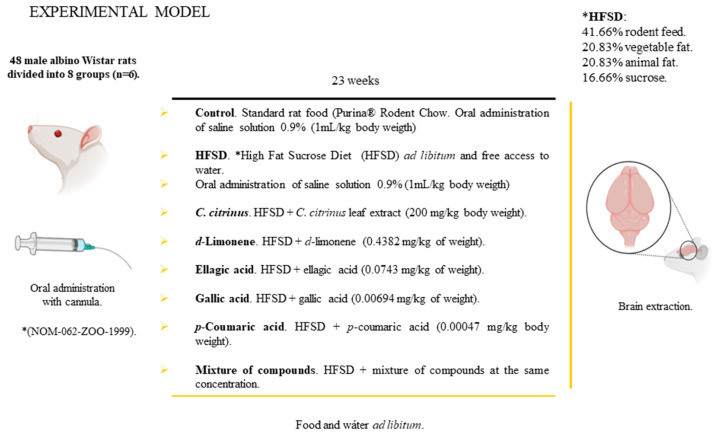
Schematic design experiment.

Figure 1 shows the experimental design: forty-eight male Wistar rats were randomly assigned to eight groups (n = 6). Group 1 served as the control group, receiving standard rodent chow (Purina^®^ Rodent Chow) and an oral dose of 0.9% saline solution at 1 mL/kg body weight. Group 2 (HFSD) was fed a high-fat, high-sucrose diet and received an oral dose 1 mL/kg of body weight of 0.9% saline solution. This high-calorie diet consisted of 41.66% Rodent Chow Purina^®^, 20.83% vegetable fat (INCA brand), 20.83% pork lard, 16.66% sucrose, and water provided ad libitum. In addition to the HFSD, group 3 received the ethanolic extract of *C. citrinus* leaves at a dose of 200 mg/kg. Groups 4, 5, 6, 7, and 8 received ellagic acid, gallic acid, p-coumaric acid, and d-limonene at doses of 0.0743 mg/kg, 0.00694 mg/kg, 0.00047 mg/kg, and 0.4382 mg/kg, respectively, along with a mixture of these compounds at the same concentrations. The sample size and doses of the various compounds are based on their concentration in the *C*. *citrinus* extract at 200 mg/kg body weight [10,11]. Food and water were ad libitum throughout the experiment. Treatments were administered once daily via oral cannula at 10:00 a.m. throughout the 23 weeks without fasting. Body weight, food intake, and water consumption were recorded daily for 23 weeks. At the end of this period, the following morphometric parameters were measured: total body weight using an electronic scale, nose-to-anus length, and nose-to-tail length using a tape measure. The adiposity index (AI), which quantifies fat accumulation, was calculated as AI = (total adipose tissue weight/final body weight) × 100. Additionally, body mass index (BMI) was calculated as kg/m^2^. In rats, the Lee index (LI) is similar to BMI in humans, calculated as LI = (3√body weight)/nose-to-anus length × 10. Weight gain (D) was calculated as D = [(final body weight − initial body weight)/initial body weight] × 100. Blood was collected for glucose and triacylglycerol analysis using Spinreact kits. After completing these measurements, the animals were euthanized with a sodium pentobarbital injection (50 mg/kg). Then, total adipose and brain tissue samples were removed, washed, weighed, and stored at −20 °C.

### 2.4. Brain Tissue Homogenate

The whole brain was homogenized in 10 mM phosphate buffer at pH 7.4, then centrifuged at 20,784× *g* for 20 min at 4 °C in an Eppendorf 5415 R centrifuge. The supernatant was collected and stored at −80 °C for evaluation of biochemical markers. Protein concentration was determined by the Bradford method [14], with bovine serum albumin as the standard.

### 2.5. Determinations of Antioxidant Enzymes and Biomarkers of Oxidative Stress in the Brain of Rats Fed a High-Calorie Diet

#### 2.5.1. Catalase (CAT)

The CAT enzyme activity was measured by monitoring the disappearance of hydrogen peroxide [15]. In quartz cells, 920 μL of 50 mM phosphate buffer at pH 7 was added, along with 30 μL of brain tissue homogenate and 50 μL of 30 mM hydrogen peroxide (H_2_O_2_). The mixture was gently shaken, and its absorbance was immediately measured in a spectrophotometer at 240 nm, with the decrease monitored over 3 min at 30-s intervals.

#### 2.5.2. Superoxide Dismutase (SOD)

The activity of the enzyme SOD was measured by tracking the production of superoxide anions, which reduce nitroblue tetrazolium chloride (NBT) to a formazan product, as described by Giannopolitis [16]. The mixture contained: 40 μL of 0.1 M ethylenediaminetetraacetic acid (EDTA), 20 μL of 1.5 mM blue nitrotetrazolium chloride (NTB), 601 μL of 50 mM phosphate buffer at pH 7.5, 50 μL of brain tissue homogenate, 9 μL of 0.1 mM riboflavin, and 0.9 μL of 10 mM methionine. The samples were gently shaken and exposed to a 40-volt lamp from a distance of 15 cm for 15 min. Finally, absorbance was measured at 560 nm using a spectrophotometer.

#### 2.5.3. Glutathione Peroxidase (GPx)

The activity of the enzyme GPx was determined as described by Prabhu [17], in a final volume of 1000 μL that contained 10 μL of 0.5 mM NADPH, 10 μL of 100 mM reduced glutathione, 4 μL of 1 U of the enzyme glutathione reductase, 901 μL of 5 mM phosphate buffer with 50 mM EDTA at pH 7, 50 μL of 30 mM cumene, and 25 μL of brain tissue homogenate. Once the mixture was prepared, the absorbance was measured at 340 nm using a spectrophotometer, with readings taken every 5 min for 5 min.

#### 2.5.4. Paraoxonase (PON1)

PON1 enzyme activity was measured as described by Dantoine [18]. A cocktail solution was prepared containing 25 mM Tris-HCl buffer at pH 8, 10 mM CaCl_2_, and 1 mM 4-nitrophenyl acetate. Then, 5 μL of brain tissue homogenate was added to 1000 μL of the prepared solution. The absorbance was measured at 402 nm over 3 min, with readings taken every 30 s.

#### 2.5.5. Reduced Glutathione (GSH)

GSH modulates cellular homeostasis and detoxifies compounds; its depletion is associated with numerous diseases [19]. GSH content was quantified as described by Sedlak and Lindsay [20]. 62.5 μL of brain tissue homogenate, 187.5 μL of 0.2 M Tris buffer, pH 8.2, and 12.5 μL of 0.01 M 5,5′-dithiobis (2-nitrobenzoic acid) (DNTB); the sample was mixed and 987.5 μL of absolute methanol was added. The samples were then placed on a laboratory shaker at 240 rpm for 15 min and afterward centrifuged at 1107× *g* at room temperature for 15 min. Finally, the absorbance of the samples was measured at 412 nm with a spectrophotometer.

#### 2.5.6. Malondialdehyde (MDA) and 4-Hydroxynonenal (4-HNE)

MDA and 4-HNE are products of membrane lipid peroxidation; these compounds contribute to many pathologies by forming adducts with macromolecules [21]. The determination of MDA and 4-HNE was conducted using the method of Johnston et al. [22]. Homogenization was performed in a cold mortar with a pestle. The mixture contained 0.5 g of brain tissue, 10 μL of 5 mM butylhydroxytoluene (BHT), and 2 mL of 10 mM phosphate buffer at pH 7.4, and was centrifuged at 15,700× *g* for 20 min at 4 °C. For the MDA assay in Eppendorf tubes, 0.65 mL of 10 mM 1-methyl-2-phenylindole dissolved in a 3:1 acetonitrile/methanol solution and 0.2 mL of brain tissue homogenate were added; 0.15 mL of 37% HCl was then added. The sample was mixed, then incubated at 45 °C for 60 min. Subsequently, the reaction was halted by placing it in an ice bath. Finally, the absorbance was measured in glass cells at 586 nm. The determination of 4-hydroxynonenal (4-HNE), as mentioned in the MDA, was conducted, except that HCl was replaced with 37% of methanesulfonic acid.

#### 2.5.7. Advanced Protein Oxidation Products (AOPP)

AOPP is used as a biomarker of oxidative stress in metabolic syndrome, which includes obesity [23]. Briefly, each sample contained 1000 μL of 20 mM phosphate buffer (pH 7.4), 50 μL of brain tissue homogenate, 50 μL of 1.16 M potassium iodide (KI), and 100 μL of acetic acid. The samples were centrifuged at 5800× *g* for 5 min, incubated for 2 min, and their absorbance at 340 nm was measured using a spectrophotometer [24].

### 2.6. Determinations of the Enzymes Involved in the Inflammatory Process in the Brain of Rats Fed a High-Calorie Diet

#### 2.6.1. Total Cyclooxygenase (COX-1 and COX-2)

The total enzymatic activity of cyclooxygenase (COX-1 and COX-2) was determined using the technique described by Kumar et al. [25]. This method employs a colorimetric assay based on the oxidation of N,N,N′,N′-tetramethyl-p-phenylenediamine dihydrochloride (TMPD) during the reduction of PGG2 to PGH2. The reaction mixture consisted of 712 µL of 100 mM Tris-HCl buffer, pH 8, 31 µL of 15 µM hematin, 31 µL of 3 µM EDTA, 100 µL of the centrifuged homogenate (at 10,000× *g* for 15 min), and 63 µL of 100 mM TMPD. After adding 63 µL of 133 µM arachidonic acid as a substrate, the mixture was combined and incubated at 25 °C for 20 min. The absorbance was then read at 590 nm. The TMPD extinction coefficient was 0.00826 µM^−1^. One enzyme unit was defined as the amount needed to oxidize one nmol of TMPD per minute.

#### 2.6.2. 5-Lipoxygenase (5-LOX)

The enzymatic determination of 5-LOX was conducted using the spectrophotometric analysis established by Kumar et al. [25]. This method involves the formation of hydroperoxides from the enzymatic reaction, during which ferrous sulfate is oxidized to ferric sulfate. During the reaction, the derived ferric sulfate can form a complex with xylenol orange, resulting in a ferric-xylenol-orange complex that exhibits a vivid blue color. The reaction mixture consisted of 490 µL of 50 mM Tris-HCl buffer at pH 7.4, 10 µL of centrifuged homogenate (centrifuged at 10,000× *g* for 5 min), and 10 µL of 133 µM arachidonic acid. After mixing, it was incubated at room temperature in the dark for 10 min. Next, 490 µL of FOX reagent was added, which contained 25 mM sulfuric acid, 100 µM orange xylenol, and 250 µM ferrous sulfate, all diluted in a water-methanol mixture (1:9). Lastly, 100 µL of butylhydroxytoluene as an antioxidant was added, mixed, and incubated as before. The final absorbance was measured at 590 nm.

#### 2.6.3. Xanthine Oxidase (XO)

XO activity was measured using the method described by Chung et al. [26]. A mixture of 40 μL of brain homogenate, 880 μL of 33 mM phosphate buffer at pH 7.5, and 100 μL of 0.17 mM xanthine was prepared. The mixture was then incubated at 37 °C for 20 min. After incubation, 200 μL of trichloroacetic acid (TCA) was added. The sample was centrifuged at 10,000× *g* for 15 min, and the resulting supernatant was measured at 293 nm using a spectrophotometer.

#### 2.6.4. Myeloperoxidase (MPO)

MPO was measured according to the method described by Suzuki et al. [27]. A mixture was prepared by combining 425 μL of 200 mM PBS at pH 5.5, 10 μL of 15 mM H_2_O_2_, and 40 μL of 20 mM tetramethylbenzidine (TMB). After mixing, 10 μL of brain homogenate was added. The mixture was incubated at 37 °C for 3 min and then cooled on ice for an additional 3 min. Finally, 1 mL of 200 mM sodium acetate at pH 3 was added, and the absorbance was measured at 655 nm for 3 min.

### 2.7. Statistical Analysis

The data were recorded and processed using Microsoft Excel 2019. Values are expressed as mean ± standard error of the mean (SEM). Statistical analysis was performed using JMP version 14.0, followed by Tukey’s multiple comparison test (a, b, c). A two-way repeated-measures ANOVA was used to analyze rat weight gain (*p* < 0.001), while a one-way ANOVA (*p* < 0.05) was applied to the remaining data analyses. Tukey’s Honestly Significant Difference (HSD) test is a post hoc test used in ANOVA to compare all possible pairs of means. When ANOVA reveals a significant difference among group means, a posthoc test, such as Tukey’s, is necessary to determine which groups differ significantly.

## 3. Results

### 3.1. Effects of an HFSD and Treatment on Body Weight

The results of the two-way repeated-measures ANOVA show that the main factor influencing the rats’ weight was growth time. In this case, F(22,123) = 87.48, *p* < 0.001, and η^2^ = 0.84, indicating that time had a highly significant effect on weight gain.

The treatment variable yielded an F(7, 123) = 131.87, *p* < 0.001, and η^2^ = 0.74, also indicated a significant effect of the treatment on weight change. Lastly, the analysis of the time-treatment interaction using statistical tests showed F(154, 123) = 5.17, *p* < 0.001, and η^2^ = 0.69, indicating a significant interaction between these two variables.

**Figure 2:**
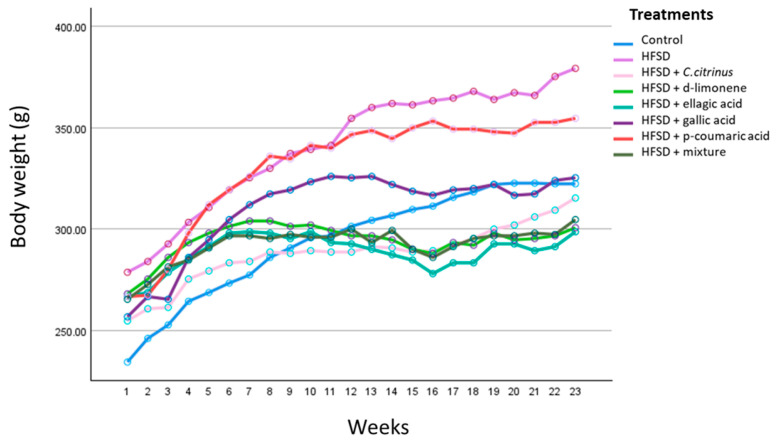
Effect of time and different treatments on body weight gain. A two-way repeated measures ANOVA was conducted to assess the impact of various treatments (8 levels) and Time (23 weeks) on rats’ weight gain.

This interaction motivates the analysis of the weekly weight gain. Figure 2 illustrates the rats’ weight over 23 weeks of study. As shown, the weight gain over time varied across treatments, and the p-coumaric acid treatment did not have a positive effect on weight gain. The control group shows continuous weight gain through week 20, after which the weight remains. The HFSD groups and those given p-coumaric acid and gallic acid display a similar pattern of weight gain through week 12, after which the slope of the curve decreases. Finally, there is a clear difference compared with the other groups: from week 7 onward, weight fluctuates little or even declines, demonstrating the effectiveness of these treatments in weight control.

Considering the interaction between time and treatment, additional statistical analyses of treatment effects were performed for each week when weight was measured. For this purpose, 23 analyses (each week) were conducted using one-way ANOVA and Tukey’s post hoc test. The results showed that, beginning in week 9, the HFSD and C. citrinus groups differed significantly. By the end of the study (week 23), the HFSD was the only group that differed significantly from the others.

In Table 1, the Lee index indicated that the group given only HFSD had a significantly higher value 338.01 ± 6.54, groups fed with HFSD and with p-coumaric and ellagic had values of 305.8 ± 10.53 and 302.46 ± 2.53. In contrast, treatments with gallic acid, p-coumaric acid, and the compound mixture showed no significant differences relative to the control group, with Lee index values that were statistically similar. Additionally, treatments with *C. citrinus* leaf extract and d-limonene resulted in Lee index values that were statistically lower than those of the control group. However, they did not differ from the values of the compound mixture. Regarding the adiposity index, the results showed that the HFSD and HFSD + ellagic acid groups had higher adiposity index values than the control group and the other treatments. However, the ellagic acid group showed adiposity index values similar to those of *C. citrinus*, d-limonene, gallic acid, p-coumaric acid, and the mixture of the compound groups (Table 1). Treatments with *C. citrinus* leaf extract, d-limonene, gallic acid, p-coumaric acid, and the compound mixtures resulted in significantly lower adiposity index values than in the HFSD group, with these differences statistically significant. However, they also exhibited statistically significant differences compared to the control group, which showed the lowest adiposity index values. Table 1 also indicates that triacylglyceride and glucose levels were higher in the HFSD group than in the control group and other treatments.

### 3.2. Effect on Antioxidant Enzymes and Oxidant Stress Biomarkers

**Figure 3:**
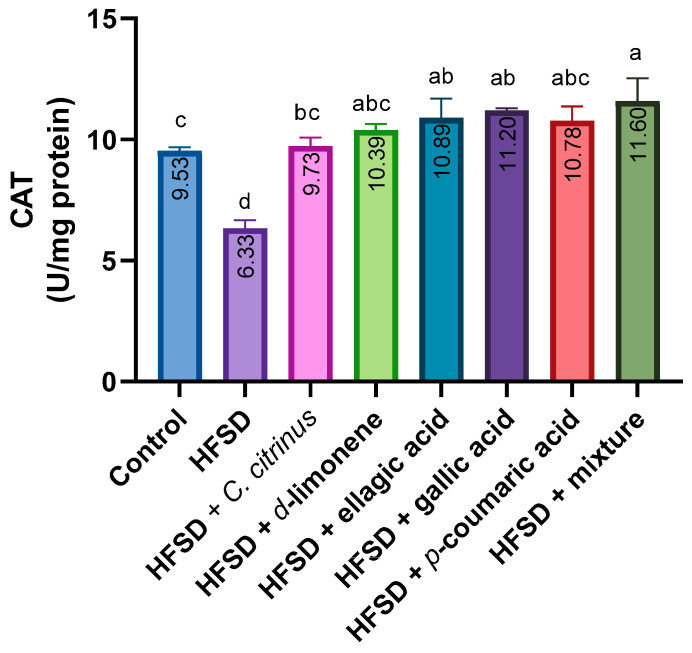
CAT activity in the brains of rats exposed to various experimental treatments. All values are expressed as mean ± SEM (n = 6). A one-way ANOVA was performed, followed by Tukey’s test (*p* < 0.05). Letters a, b, c, and d indicate statistically significant differences between the control and HFSD groups. O’Brien test *p* = 0.40.

The enzymatic activity of CAT in rat brains showed a statistically significant decrease in the HFSD group compared to other groups. Meanwhile, HFSD supplementation with *C. citrinus*, d-limonene, and p-coumaric acid resulted in a slight increase in CAT activity compared with the control group. However, combining HFSD with the mixture of ellagic acid and gallic acid resulted in a significant increase in activity relative to the control group (Figure 3).

**Figure 4:**
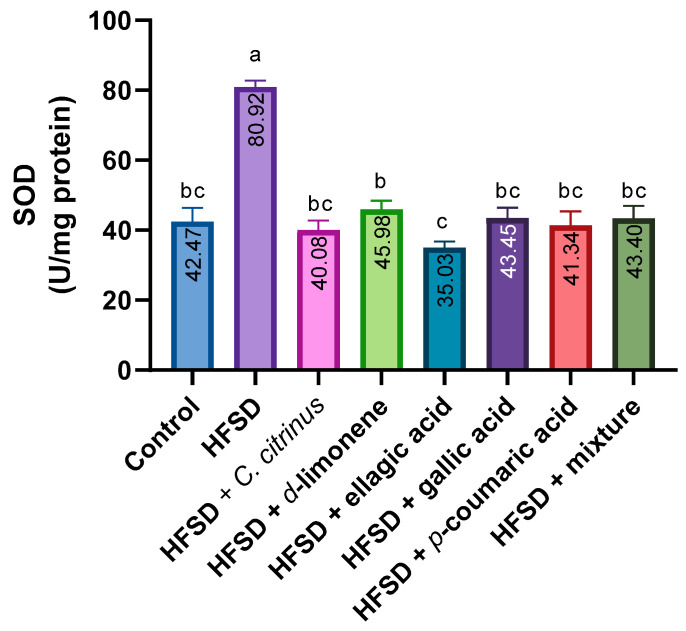
SOD activity in the brains of rats exposed to various experimental treatments. All values are expressed as mean ± SEM (n = 6). A one-way ANOVA was performed, followed by Tukey’s test (*p* < 0.05). Letters a, b, and c denote statistically significant differences between the control and HFSD groups. O’Brien test *p* = 0.86.

The results showed that SOD activity in the brain tissue of the HFSD group was higher than in the other groups (Figure 4). On the other hand, HFSD supplementation with d-limonene and ellagic acid differed between the two groups, whereas the groups remained similar to the control group.

**Figure 5:**
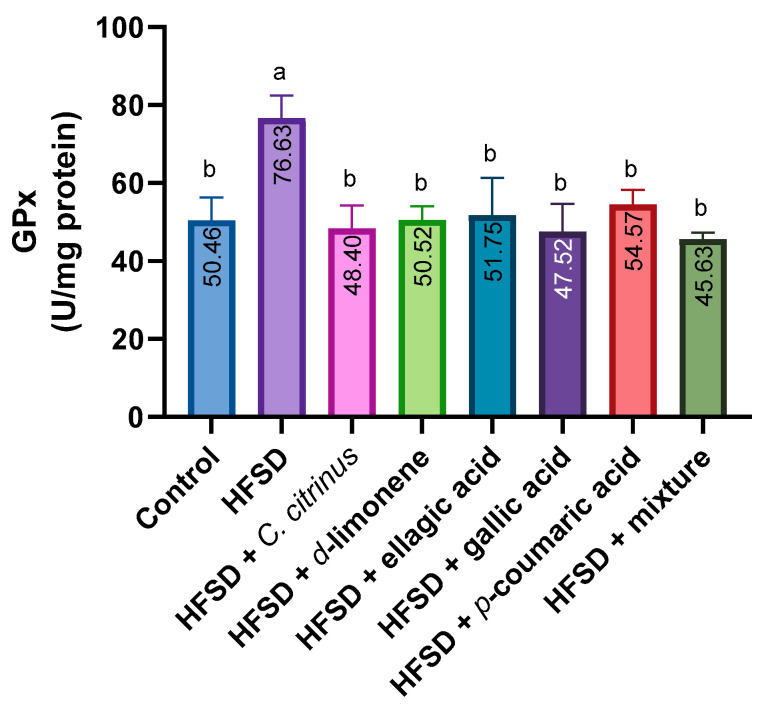
GPx activity in the brains of rats exposed to various experimental treatments. All values are expressed as mean ± SEM (n = 6). A one-way ANOVA was performed, followed by Tukey’s test (*p* < 0.05). Letters a and b denote statistically significant differences between the control and HFSD groups. O’Brien test *p* = 0.62.

GPX activity in the HFSD group increased significantly compared with the control group. Additionally, the enzyme activity levels in the other treatments did not differ from those in the control group (Figure 5).

**Figure 6:**
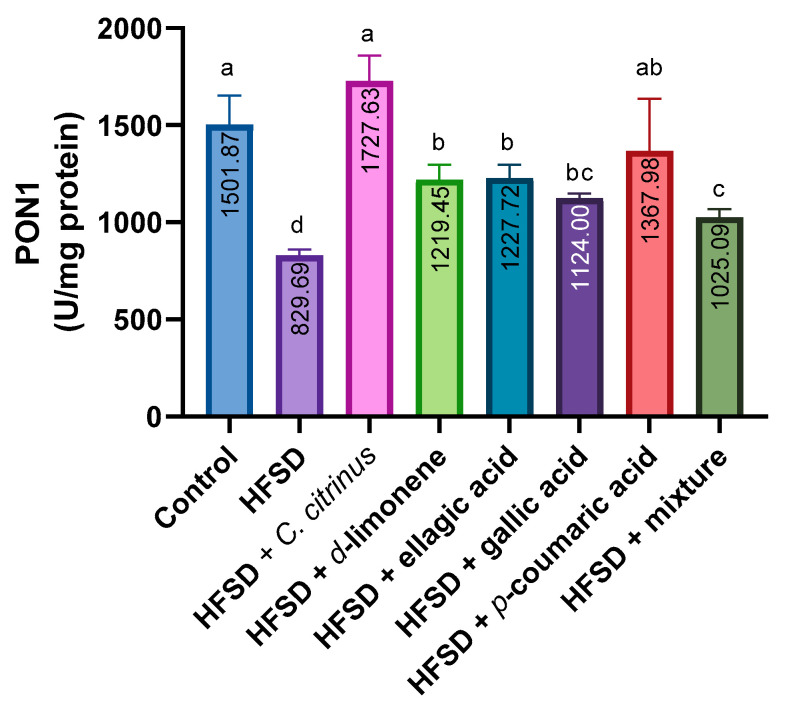
PON1 activity in the brains of rats exposed to various experimental treatments. All values are expressed as mean ± SEM (n = 6). A one-way ANOVA was performed, followed by Tukey’s test (*p* < 0.05). Letters a, b, c, and d denote statistically significant differences between the control and HFSD groups. O’Brien test *p* = 0.28.

PON1 activity was lowest in the HFSD group compared with the other groups. The HFSD treated with *C. citrinus* exhibited a greater increase in GPX activity than the other treated groups. Interestingly, the groups that received d-limonene and ellagic acid showed a slight increase in PON1 activity. In the mixture group, the activity was low but higher than in the HFSD group (Figure 6).

**Figure 7:**
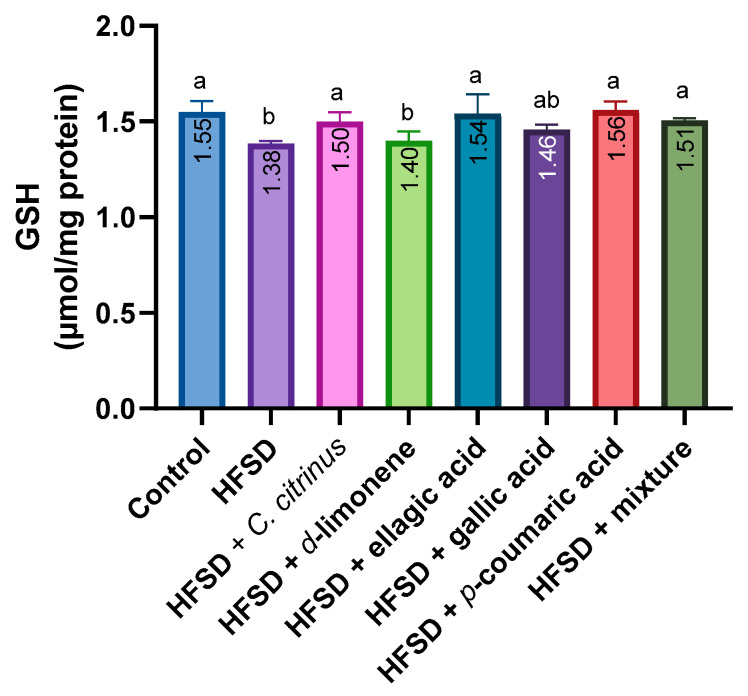
GSH levels in the brains of rats exposed to various experimental treatments. All values are expressed as mean ± SEM (n = 6). A one-way ANOVA was performed, followed by Tukey’s test (*p* < 0.05). Letters a and b denote statistically significant differences between the control and HFSD groups. O’Brien test *p* = 0.37.

Regarding oxidative stress biomarkers in the rat brain, GSH content was significantly lower in the HFSD and HFSD plus d-limonene groups than in the control, HFSD plus *C. citrinus*, ellagic acid, p-coumaric acid, and mixture groups. In contrast, the gallic acid group had GSH levels comparable to those of the control and HFSD groups (Figure 7).

**Figure 8:**
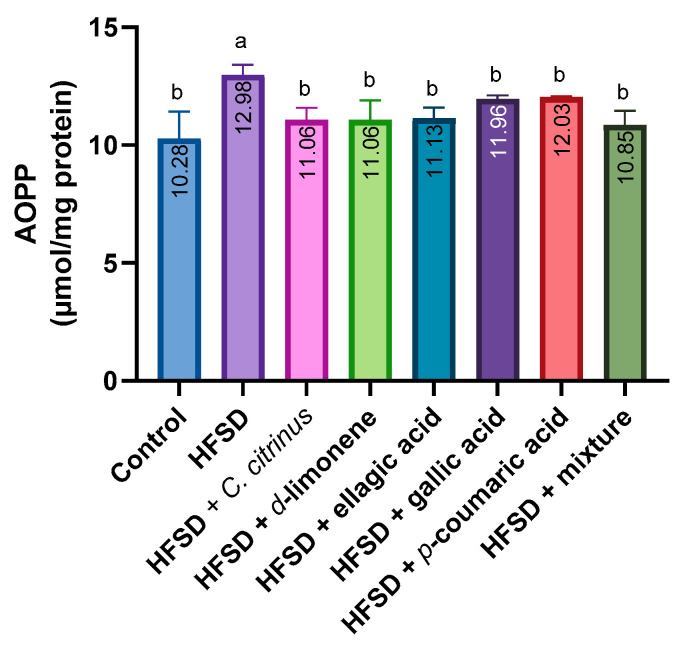
AOPP activity in the brains of rats exposed to various experimental treatments. All values are expressed as mean ± SEM (n = 6). A one-way ANOVA was performed, followed by Tukey’s test (*p* < 0.05). Letters a and b denote statistically significant differences between the control and HFSD groups. O’Brien test *p* = 0.42.

Regarding AOPP content, the results showed a significant increase in the HFSD group compared with all other groups (Figure 8).

**Figure 9:**
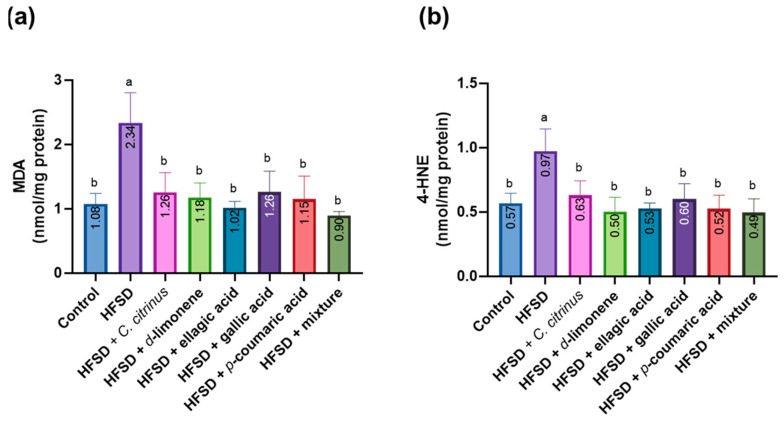
MDA (**a**) and 4-HNE (**b**) levels in the brains of rats subjected to various experimental treatments. All values are expressed as mean ± SEM (n = 6). A one-way ANOVA was performed, followed by Tukey’s test (*p* < 0.05). Letters a and b denote statistically significant differences between the control and HFSD groups. O’Brien test in MDA *p* = 0.56; O’Brien test in 4-HNE *p* = 0.78.

The results for lipid peroxidation products show that the HFSD group exhibited a significant increase in MDA (Figure 9a) and 4-HNE (Figure 9b) levels compared with the control group. The other groups fed a high-fat-sucrose diet, and those treated with *C. citrinus* extract, d-limonene, ellagic acid, p-coumaric acid, gallic acid, and the mixture showed levels comparable to those of the control group.

### 3.3. Effect of C. citrinus and Its Compounds on Pro-Inflammatory Enzymes

**Figure 10:**
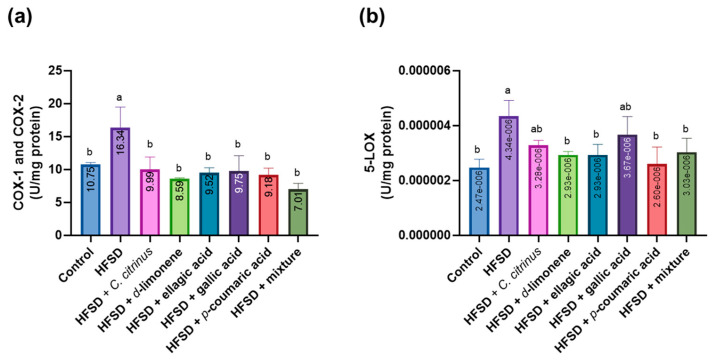
Cyclooxygenase (COX-1 and COX-2) (**a**) and 5-LOX (**b**) levels in the brains of rats subjected to various experimental treatments. All values are expressed as mean ± SEM (n = 6). A one-way ANOVA was performed, followed by Tukey’s test (*p* < 0.05). Letters a and b denote statistically significant differences between the control and HFSD groups. O’Brien test in COX-1 *p* = 0.35; O’Brien test in 5-LOX *p* = 0.68.

The total cyclooxygenase activity (COX-1 and COX-2) in the HFSD group showed a statistically significant increase compared to the control group. All other groups had significantly lower activity levels than the HFSD group, but their activity levels were statistically similar to those of the control group (Figure 10a). On the other hand, the HFSD group exhibited a significant increase in 5-LOX activity compared to the control group. Meanwhile, the groups treated with d-limonene, ellagic acid, p-coumaric acid, and the mixture of compounds displayed values similar to the control group. Additionally, they showed statistically comparable levels to the HFSD group. In the *C. citrinus* and gallic acid groups, values were similar to those of the HFSD groups, but the latter also shared characteristics with the other treated groups (Figure 10b).

**Figure 11:**
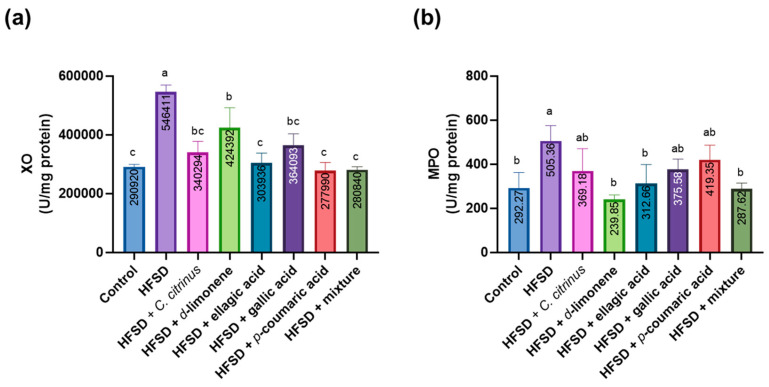
XO (**a**) and MPO (**b**) levels in the brains of rats subjected to various experimental treatments. All values are expressed as mean ± SEM (n = 6). A one-way ANOVA was performed, followed by Tukey’s test (*p* < 0.05). Letters a, b, and c denote statistically significant differences between the control and HFSD groups. O’Brien test in XO *p* = 0.41; O’Brien test in MPO *p* = 0.69.

The XO enzyme results showed a significant increase in the HFSD group compared to the other groups. The d-limonene group had notably higher activity than the control group, but its activity was lower than that of the HFSD group. Meanwhile, the groups with ellagic acid, p-coumaric acid, and the mixture of compounds showed enzyme activity similar to the control group, with no significant difference. On the other hand, the *C. citrinus* and gallic acid groups had values identical to both the control and the other treated groups (Figure 11a). Finally, MPO activity increased only in the HFSD group. In contrast, groups treated with d-limonene, ellagic acid, and the mixture showed MPO activity comparable to the control group. However, the *C. citrinus*, p-coumaric, and gallic acid groups did not show significant differences from either the HFSD or the control groups (Figure 11b).

## 4. Discussion

Our study demonstrates, for the first time, the effect of the main compounds of *C. citrinus* on oxidative stress in rat brains induced by HFSD consumption. Measuring CAT, SOD, and GPx activity is a key indicator of oxidative stress, as these enzymes are the main antioxidants that directly neutralize ROS [28]. Therefore, their activity can serve as a direct indicator of the organism’s oxidative stress state. This study showed a decrease in CAT activity in the group treated with HFSD alone. These results align with those reported by De Mello et al. [29], who examined brain changes in mice within a model of obesity induced by a high-fat diet. They found that animals in the HFD group showed reduced CAT enzyme activity and increased SOD levels in the hypothalamus, hippocampus, prefrontal cortex, and striatum. In another study by Maciejczyk et al. [30], who assessed oxidative damage in the hypothalamus and cerebral cortex of rats with insulin resistance caused by a high-fat diet, they observed an increase in SOD-1 activity in the cerebral cortex of the HFD group.

Our findings reveal a decrease in CAT activity along with an increase in SOD and GPx activity, indicating a potential oxidative stress condition in the brains of rats in the HFSD group. This is evidenced by higher activity of SOD and GPx, driven by increased ROS levels, mainly resulting from greater superoxide anion production. Additionally, the rise in superoxide anion generation is also influenced by SOD activity, while CAT and GPx help reduce it through dismutation. In this context, we conclude that the increased GPx activity in the HFSD group is linked to higher SOD activity and lower GSH levels.

On the other hand, the fact that our results showed enzyme activity levels similar in the groups treated with *C. citrinus* leaf extract, d-limonene, gallic acid, p-coumaric acid, and ellagic acid alone or in combinations suggests that these compounds are responsible for the antioxidant activity of this plant, as reported in earlier studies [10,11]. These metabolites may reduce oxidative stress by decreasing the activity of enzymes such as SOD and GPx, while increasing CAT in the brains of rats on a hypercaloric diet.

PONs consist of three isoforms: PON1 and PON3, which are produced in the liver and found in serum, where they bind to HDL to prevent LDL oxidation [31]. PON2 is absent from serum but has been identified in several tissues [32]. PONs can regulate oxidative stress (OS) production and exhibit anti-inflammatory effects [33]. Low PON1 activity has been linked to a potential risk factor for neurological disorders, including Alzheimer’s disease [34].

Francik et al. [35] reported that diets high in fructose and fat decrease PON1 activity in rat brain tissue. Conversely, Ashara et al. [36] reported low PON1 levels in mice with cerebral ischemia, and intravenous administration of PON1 reduced brain damage. In our study, PON1 activity is reduced in the brain under HFSD, which may be related to brain damage. However, the groups that received *C. citrinus* leaf extract, d-limonene, ellagic acid, and p-coumaric acid showed PON1 activity levels similar to those of the control group, despite consuming HFSD. These results suggest that consuming plant foods rich in bioactive compounds, such as polyphenols and terpenes, may help regulate antioxidant enzyme activity during oxidative stress.

We also observed a decrease in GSH content, accompanied by increases in AOPP, MDA, and 4-HNE, in the group treated only with HFSD. This indicates a positive response, consistent with oxidative stress caused by a diet high in fat and sugar. These findings agree with those reported by Keshk et al. [37], who observed that Wistar rats fed an HFD exhibited a significant decrease in GSH and a notable increase in MDA levels in the cerebral cortex. Similarly, a study conducted by De Mello et al. [29] reported decreases in GSH levels and increases in MDA levels in the hypothalamus, hippocampus, prefrontal cortex, and striatum of mice on a high-fat diet. These findings are consistent with ours.

Although different experimental treatments affected each oxidative stress biomarker in distinct ways, the HFSD combined with ellagic acid, p-coumaric acid, and the mixture groups effectively improved all biomarkers, returning them to levels comparable to those of the control group.

The antioxidant capacity of ellagic acid in the brain has been demonstrated in various oxidative stress models. Uzar et al. [38] investigated the effect of ellagic acid on oxidative stress in streptozotocin-induced diabetic rats. The results showed that diabetic rats had significantly lower PON-1 and CAT activities and higher MDA levels in brain tissue. Treatment with ellagic acid in diabetic rats notably restored PON-1 and CAT activities and reduced MDA levels. Another study [39] found that ellagic acid increased CAT activity and GSH levels while lowering MDA levels in the brains of rats with CCl_4_-induced injury.

The effects of p-coumaric acid on oxidative stress conditions have also been documented. Sakamula et al. [40] reported that pretreatment with p-coumaric acid significantly reduced MDA levels while also increasing the activity of CAT and SOD in the brains of mice with ischemia–reperfusion injury. Conversely, the effect of limonene was tested in a rat model of dopaminergic neurodegeneration induced by rotenone. It was found that treatment with limonene significantly reduced MDA levels and substantially increased SOD and CAT activities, as well as GSH concentrations, in the rat midbrain after rotenone injection, compared with rotenone-injected rats [41].

We observed increased activity of total cyclooxygenase (COX-1 and COX-2), 5-LOX, XO, and MPO in the group fed only HFSD. This study highlights the inflammatory response in the brains of rats caused by a high-fat, high-sugar diet. Conversely, we observed different effects of *C. citrinus* leaf extract, d-limonene, ellagic acid, gallic acid, p-coumaric acid, and their combination on various inflammatory enzymes. The most effective treatments, including HFSD + ellagic acid, HFSD + p-coumaric acid, and the combination of all four compounds, showed positive effects on most enzymes. Eddin et al. [41] found that treating rats with limonene after rotenone injection significantly reduced COX-2 expression in the rats’ striatum. This finding aligns with ours, in which we observed a notable decrease in the activity of total cyclooxygenase (COX-1 and COX-2) in the brains of rats fed an HFSD and administered d-limonene.

MPO is an enzyme found in immune cells and serves as a biomarker for neutrophil infiltration. MPO generates hypochlorous, hypobromous, and hypothiocyanous acids through the oxidation of H_2_O_2_ [42]. Gellhaar et al. [43] reported that MPO levels are increased in the brains of Parkinson’s and Alzheimer’s patients. Althurwi et al. [44] demonstrated that β-carotene decreased MPO activity in the brains of rats with ischemic/reperfusion injury. A significant increase in MPO enzyme activity was observed in the cerebral cortex of Wistar rats fed a high-fat diet (HFD) [31]. This finding aligns with the results reported in the current study. It has already been shown that some tissues of rodents fed high-fat, high-carbohydrate diets exhibit impaired redox homeostasis, including elevated activity of enzymes that generate free radicals, such as XO and MPO [45].

Our study found that treatments with *C. citrinus* extract, d-limonene, ellagic, gallic, and p-coumaric acids, as well as a mixture of these compounds, significantly reduced the activity levels of both enzymes, even when HFSD was consumed. Interestingly, the groups fed an HFSD and supplemented with these compounds showed a beneficial effect. These results align with reports that these compounds significantly enhance the expression of antioxidant enzymes and decrease the levels of oxidative stress biomarkers and pro-inflammatory enzyme activities in other tissues [11].

This study demonstrated that d-limonene and the gallic, p-coumaric, and ellagic acids are the compounds responsible for the antioxidant and anti-inflammatory effects of *C. citrinus* extract in the brains of Wistar rats fed an HFSD. The limitation of this study was the small number of animals per group (n = 6). According to the Animal Bioethical Committee of UMSNH, studies involving animals must minimize the number of animals used. The number n = 6 is the minimum considered sufficient for all enzymatic and biomarker assays. However, it restricts the use of different brain regions for histopathological analysis. Additionally, administering substances via a cannula could cause esophageal discomfort and stress, potentially leading to weight loss independent of the therapeutic effect. In this study, both the control group and the group fed a high-fat-sucrose diet also received water through a cannula to ensure that all groups were exposed to the same stressor. Other limitations of our study included the analysis of only one tissue type, one age group, and one period of obesity. It is essential to conduct studies that include more tissues, various ages, different durations of obesity, and female rodents. Finally, despite the promising results of this study, before considering human use, additional studies should be conducted that employ superior animal models.

## 5. Conclusions

This study demonstrates that extracts of *C. citrinus*, d-limonene, ellagic acid, and their combinations have anti-obesity effects. In contrast, p-coumaric acid did not show such effects. Moreover, administering ellagic acid, p-coumaric acid, and the mixture increased antioxidant enzyme activities, such as SOD, PON, and GSH, while decreasing the activities of 5-LOX, XO, and MPO to levels comparable to those of the control group. On the other hand, *C. citrinus* extract and its compounds attenuated AOPP, MDA, and 4-HNE levels, as well as COX activity, in a similar manner. Interestingly, the levels of these compounds are lower than those reported in other studies. Our findings support the beneficial effects of *C. citrinus* extract against obesity and oxidative stress. However, research on the pro-inflammatory cytokines and signaling pathways involved in the compounds present in *C. citrinus* would help clarify the mechanism of action of *C. citrinus* extract. Future studies should analyze other compounds, such as 1,8-cineole and α-terpineol, found in *C. citrinus*, for a more complete understanding of the additional biological activities reported in *C. citrinus*.

## Figures and Tables

**Table 1:** Morphometric and biochemical parameters of Wistar rats from different experimental treatments.

	Control	HFSD	HFSD + *C. citrinus*	HFSD + *d*-Limonene	HFSD + Ellagic Acid	HFSD + Gallic Acid	HFSD + *p*-Coumaric Acid	HFSD + Mixture
**Final weight (g)**	322.33 ± 10.34 c	379.33 ± 37.94 a	315.33 ± 24.50 c	300.66 ± 15.97 c	298.66 ± 10.34 c	325.33 ± 12.50 bc	354.66 ± 29.11 ab	304.66 ± 5.73 c
**Weight gain (%)**	32.45 ± 20.17 b	57.33 ± 28.72 a	33.98 ± 1.44 b	31.17 ± 9.29 b	32.54 ± 18.58 b	27.73 ± 6.33 b	54.05 ± 5.95 a	26.78 ± 6.48 b
**Lee index**	298.36 ± 11.08 bc	338.01 ± 6.54 a	282.35 ± 10.62 de	280.41 ± 9.05 e	302.46 ± 2.53 bc	295.69 ± 8.34 bcd	305.84 ± 10.53 b	289.17 ± 3.61 cde
**Adiposity index**	2.85 ± 1.57 c	8.95 ± 2.13 a	6.25 ± 2.00 b	5.21 ± 1.99 b	7.02 ± 3.52 ab	5.99 ± 1.52 b	6.48 ± 0.56 b	6.17 ± 1.37 b
**Triacylglycerides (mg/dL)**	71.16 ± 9.92 b	177.05 ± 9.91 a	39.69 ± 9.92 c	40.93 ± 9.92 c	58.91 ± 9.91 bc	65.58 ± 9.92 b	59.84 ± 9.91 bc	58.91 ± 9.91 bc
**Glucose (mg/dL)**	89.33 ± 2.87 c	109.33 ± 3.79 a	99.66 ± 14.56 abc	92.33 ± 9.40 bc	92 ± 16.29 bc	85.66 ± 13.68 c	104.33 ± 7.17 ab	85.33 ± 21.13 c

Values presented as mean ± SD (n = 6; Tukey’s post hoc test; values are statistically significant at *p* < 0.05). Letters a, b, c, d and e indicate statistically significant differences between the control and HFSD groups.

## Data Availability

The raw data supporting the conclusions of this article will be made available by the authors on request.
